# Programmed Intermittent Epidural Boluses of 0.1% Ropivacaine Versus 0.2% Ropivacaine for the Maintenance of Epidural Analgesia in Labor

**DOI:** 10.7759/cureus.63564

**Published:** 2024-07-01

**Authors:** Emmanouil Stamatakis, Konstantina Panagouli, Sophia Hadzilia, Michail Pavlidis, Vassiliki Skandalou, Anastasia Loukeri, Athanasia Saiti, Dimitrios Valsamidis

**Affiliations:** 1 Department of Anesthesiology, Alexandra General Hospital of Athens, Athens, GRC; 2 Second Department of Anesthesiology, Attikon University General Hospital, Athens, GRC

**Keywords:** motor blockade, ropivacaine, pieb, labor, epidural

## Abstract

Objective

The gold standard for pain management during labor is epidural analgesia, which can be administered in two different ways to the parturients, either by bolus doses or continuous infusions of local anesthetic solutions with opioids. Recently, programmed intermittent epidural boluses (PIEBs) via a pump are gaining popularity as a very effective method with minimal side effects. The aim of this study was to evaluate the optimum ropivacaine concentration between two different regimens (0.1% or 0.2% both with fentanyl 2 μg/ml) that can provide satisfactory analgesia with the minimum degree of motor blockade, using PIEBs.

Methods

A prospective randomized controlled study was performed from March 2020 to March 2022. Two different concentrations of ropivacaine 0.1% and 0.2% via PIEBs were equally allocated to two groups of parturients with an additional patient control epidural rescue bolus if needed. Our primary endpoint was motor blockade, as assessed by the modified Bromage scale (MBS). We also recorded visual analog scale (VAS) scores, heart rate, blood pressure, total local anesthetic consumption, labor duration and method of delivery, and APGAR score of the newborns.

Results

All patients presented Bromage scores equal to 6, and the total consumption of the anesthetic solution was comparable between the two groups. Women in the 0.2% group showed higher pain relief and satisfaction compared to the 0.1% group. Concerning the 0,2% group, diastolic blood pressure and APGAR scores were lower alongside with a lower satisfactory extrusion stage observed by the obstetrician.

Conclusion

Both ropivacaine regimens provide satisfactory labor epidural analgesia for the expectant mother without any motor blockade.

## Introduction

Labor can be a very painful process for the mother, which comes in contrast with the joy of having a newborn baby, and is often described as the worst pain a woman will experience in her lifetime. Modern medicine has made a lot of progress toward tackling this problem. Nowadays, standard practice for labor consists of placing an epidural catheter in the laboring parturient and administrating dilute solutions of local anaesthetics, such as bupivacaine or ropivacaine with or without the addition of opioids, for pain relief [1]. Administration of local anaesthetics can be achieved either by continuous infusion or by intermittent boluses. Intermittent boluses may provide a more uniform spread of the local anaesthetic in the epidural space and subsequently better sensory block. Hogan et al. [2] studied the spread of ink in cadaveric epidural spaces and found that the most uniform spread can be achieved by administrating high volumes of ink with high pressures. An experiment by Kaynar and Shankar [3] found that intermittent boluses rather than continuous infusions provided a greater spread of isotonic methylene blue through a multi-orifice catheter. This evidence suggests that the administration of local anaesthetics by intermittent boluses generates greater pressures in the epidural space and thus greater spread. Such results seem to be confirmed by other studies [4].

The literature suggests that programmed intermittent epidural boluses (PIEBs) are more effective in the pain management of abdominal and gynecologic surgery [5-6]. Moreover, the use of PIEBs during labor is linked to fewer adverse effects, such as motor blockade and prolonged duration of labor, and it provides better patient satisfaction [7-9]. This evidence encouraged us to change our local protocols, which consisted of on-demand boluses of 0.2% ropivacaine with fentanyl, and gave us the initiative to investigate if we could achieve similar results in our clinical setting. For this purpose, we conducted a prospective, randomized, controlled study comparing PIEB of 0.2% ropivacaine with PIEB of 0.1% ropivacaine to evaluate the optimal concentration for alleviating pain with minimal side effects. To our knowledge, there is not a comparison between 0.1% and 0.2% ropivacaine with fentanyl, using the PIEB method for labor in the literature.

## Materials and methods

From March 2020 to March 2022, a prospective randomized controlled study was conducted in Alexandra General Hospital of Athens in Greece. This trial was approved by the Scientific Council and Ethics Committee of the hospital (156/17/03/2020 subj 73/27/05/2020), and all the participants provided written consent for enrolment according to the Declaration of Helsinki standards.

A total of 104 American Society of Anesthesiologists (ASA) I parturients scheduled for vaginal labor were recruited for this study, between 18 and 45 years of age. Inclusion criteria were nulliparous or para 2, singleton pregnancy, gestational age above 38 weeks, body mass index (BMI) below 30(before pregnancy), and cephalic presentation of the fetus. Exclusion criteria were gestational diabetes treated with insulin and any other pathology of the pregnancy, such as amniotic fluid disorders, gestational hypertension and cervical incompetence, any contraindication for epidural placement, such as the use of anticoagulants without previous termination and skin infection at the site of placement, known allergy to the administrated medications, cervical dilation greater of 6 cm, and the inability to comprehend Greek.

Four participants did not provide consent and were excluded.

In the delivery room, the parturients were monitored with continuous electrocardiography, pulse oximetry, and noninvasive blood pressure measurement every 15 minutes. All parturients had an 18G peripheral venous catheter inserted. Epidural catheter placement was performed in the sitting or lateral decubitus position, at the L2-L3 or L3-L4 vertebral interspace using the loss of resistance to air technique with an 18G Tuohy needle and a multipored catheter. Once the epidural catheter was inserted, a test dose of 2% lidocaine 2 ml was given via the catheter to confirm the correct placement. After five minutes, the programmed intermittent epidural bolus pump (Rythmic^TM^ Evolution Micrel Medical Devices SA) was commenced, providing an initial 10 ml bolus dose followed by an 8 ml programmed bolus after one hour and subsequent doses delivered in one-hour intervals. In addition, if needed, the parturient could receive on demand a 5 ml bolus dose of the solution with a 20-minute lockout interval.

Drops in the mean arterial pressure below 70 mmHg were resolved by the administration of 5 mg boluses of ephedrine or 250 ml Ringer's lactate infusion, according to the anesthetist's preference.

All women were randomly allocated to the study group using a computer-generated list, with an allocation ratio of 1:1. Allocation concealment was achieved using opaque sealed envelopes; an anesthetist not involved in data collection programmed the epidural pump after the epidural was secured. Group A50 consisted of 50 women who were assigned to 0.1% ropivacaine with fentanyl 2 mcg/ml and group B50 consisted of 50 women who were assigned the 0.2% ropivacaine solution also enriched with fentanyl 2 mcg/ml. The maximum permissive epidural dose per hour was 23 ml in both groups.

Data recorded were motor blockade as defined by the Modified Bromage Score (MBS) (Table 1) every hour, non-invasive blood pressure and heart rate every 15 minutes, pain measured by the visual analog scale (VAS, 0 = no pain, 10 = worst pain experienced) every 30 minutes, and APGAR score of the newborn in the first, fifth, and 10th minutes. In addition, overall consumption of the ropivacaine-fentanyl solution, first and second stages of labor duration, method of delivery (vaginal, instrumental, and cesarean section) and the obstetrician feedback regarding the efficiency of the parturients' pushing during delivery were also recorded.

**Table 1:** Modified Bromage score

Score	Definition
1	Complete motor blockade
2	Almost complete motor blockade
3	Partial motor blockade, patient able to move the knees
4	Detectable weakness of hip flexion, patient able to raise leg but cannot keep raised
5	No detectable weakness of hip flexion, patient able to raise leg for 10 seconds
6	No weakness

The primary endpoint was the degree of motor blockade assessed by the MBS. Secondary endpoints were the total consumption of ropivacaine, the duration of the first and second stages of labor, the method of delivery of the fetus, the pain of the parturient expressed by VAS score, and the efficacy of parturients' pushing during the extrusion stage based on the obstetricians' records.

Quantitative variables were expressed as mean (standard deviation) or as median (interquartile range) values. Qualitative variables were expressed as absolute and relative frequencies. Students’ t-tests and Mann-Whitney tests were used for the comparison of continuous variables between the two groups. For the comparison of proportions, chi-square and Fisher’s exact tests were used. Data normality was determined with the Mann-Whitney test. Data were modeled using mixed linear models with dependent variables participants’ SAP, DAP, and VAS scores. Adjusted regression coefficients (β) with standard errors (SE) were computed from the results of the mixed models. Mixed linear models with dependent variables participants’ VAS scores were done using the logarithmical transformation of the VAS scale. All reported p-values are two-tailed. Statistical significance was set at p < 0.05, and analyses were conducted using Stata Statistical Software release 13 (2013, StataCorp, College Station, TX: StataCorp LLC).

## Results

Our sample consisted of 100 women, divided into two equally sized groups of 50 each. Their characteristics are presented by group in Table 2.

**Table 2:** Sample characteristics, by group a. Student’s t-test, b. Pearson’s chi square test

Characteristics	Α50 (N = 50)	B50 (N = 50)	P value
	Mean (SD)	Mean (SD)	
Age (years)	30.6 (4.7)	30 (5.2)	0.548^a^
Height (m)	1.7 (0.1)	1.6 (0.1)	0.151^a^
Weight (kg)	62.2 (9.1)	63.3 (8.6)	0.558^a^
BMI (kg/m^2^)	22.7 (2.7)	23.8 (4.0)	0.109^a^
Gestational age (weeks)	39.6 (0.9)	39.2 (1.3)	0.112^a^
Parity, N (%)			
Nulliparous	37 (74.0)	34 (68.0)	0.509^b^
Multiparous	13 (26.0)	16 (32.0)	

No significant differences were found between the two groups. Information on participants’ anesthesia in each group is presented in Table 3.

**Table 3:** Information on the participants’ anesthesia, by group a. Mann-Whitney test, b. Pearson’s chi-square test

	Α50	B50	P value
	N	N	
Oxytocin			
No (%)	23 (46.0)	16 (32.0)	0.151^b^
Yes (%)	27 (54.0)	34 (68.0)	
ASA, median (IQR)	1 (1─1)	1 (1─1)	0.240^a^
Cervix dilation (cm), median (IQR)	2 (2─3)	2 (2─3)	0.897^a^
Epidural space, N (%)			
Ο2-3	16 (32.0)	20 (40.0)	0.405^b^
Ο3-4	34 (68.0)	30 (60.0)	
Solution consumption (ml), median (IQR)	41 (31─59)	43 (26─58)	0.689^a^
Total number of doses by request, median (IQR)	2 (1─3)	1 (0─1)	<0.001^a^

The doses of oxytocin that were administrated were not significantly different between the two groups. Parturients in both groups were presented with comparable cervical dilation of 2 cm. The total volume of solutions that were infused was almost the same. Specifically, in group A50, the volume infused was 41 ml, and in group B50, it was 43 ml. The number of doses requested by the participants was higher in the A50 group; more specifically, two additional doses were given in the A50 group compared to one dose in the B50 group. This difference was proven statistically significant.

Information on the participants’ labor is presented by group in Table 4.

**Table 4:** Information on the participants’ labor and delivery, by group a. Student’s t-test, b. Mann-Whitney test, c. Pearson’s chi-square test, d. Fisher’s exact test

	Α50	B50	P value
	N	N	
Birth weight (gr), mean (SD)	3290.5 (283.3)	3290.5 (154.7)	>0.999^a^
Child gender			
Male (%)	35 (70.0)	30 (60.0)	0.295^c^
Female (%)	15 (30.0)	20 (40.0)	
APGAR 1 min, median (IQR)	9 (8─9)	7.2 (7.2─9)	0.001^b^
APGAR 5 min, median (IQR)	9 (9─9)	8.6 (8.6─9)	<0.001^b^
APGAR 10 min, median (IQR)	9.2 (9─9.2)	8.9 (8.9─8.9)	<0.001^b^
Forceps delivery			
No (%)	48 (96.0)	43 (86.0)	0.160^d^
Yes (%)	2 (4.0)	7 (14.0)	
Cesarean section			
No (%)	40 (80.0)	43 (86.0)	0.424^c^
Yes (%)	10 (20.0)	7 (14.0)	
Duration of labor (min), mean (SD)	205.8 (98.5)	318.9 (168.9)	<0.001^a^
Duration of 1st stage (min), median (IQR)	209.7 (209.7─209.7)	499.3 (360─499.3)	<0.001^b^
Duration of 2nd stage (min), median (IQR)	44.1 (35─44.1)	45.8 (35─45.8)	0.016^b^

**Figure 1:**
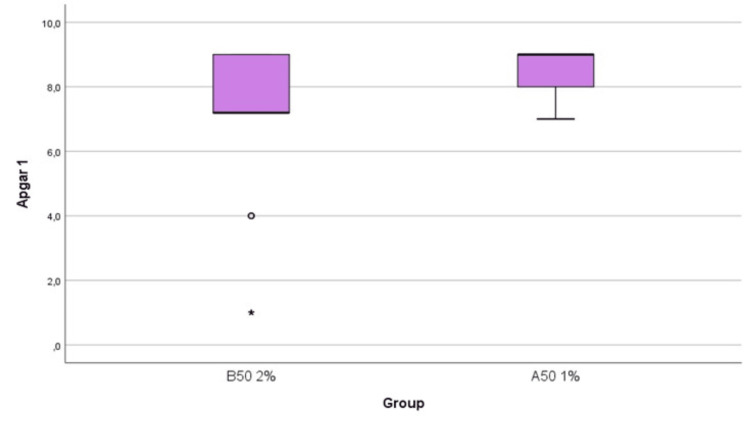
APGAR score at the first minute, by group *: extreme value, ◦: outlier

The APGAR score at one minute was significantly lower in the B50 group, 9 versus 7.2 (Figure 1), as well as the APGAR scores at five and 10 minutes, respectively. There was no statistically significant difference in instrumental delivery via forceps between the two groups; however, the absolute number was higher in the B50 group (seven deliveries in comparison with two deliveries in the A50 group). Moreover, cesarean section delivery incidence was comparable in both groups, with a higher absolute number in the A50 group. The duration of the first stage of labor was significantly higher in the B50 group with 499.3 minutes of median duration versus 209.7 minutes in the A50 group. Total labor duration was longer in the B50 group (318.9 minutes in the B50 group vs. 205.8 minutes in the A50 group). By contrast, the second stage of labor presented a similar duration between the two groups (44.1 minutes in the A50 group vs. 45.8 minutes in the B50 group).

The systolic arterial pressure (SAP) did not change significantly throughout the follow-up period and did not differ significantly between the two groups (Table 5).

**Table 5:** Results from the mixed linear regression models for SAP, DAP, and VAS a. regression coefficient, b. standard error, c. conducted after the logarithmic transformation of the visual analog scale (VAS) SAP: systolic arterial pressure, DAP: diastolic arterial pressure

Dependent variables	Independent variables	β^a^	SE^b^	P-value
SAP	Time	0.29	0.37	0.416
	Group (B50 vs. Α50)	-4.28	5.25	0.415
	Time and group interaction term	-0.54	0.45	0.225
DAP	Time	-0.41	0.06	<0.001
	Group (B50 vs. Α50)	-4.66	1.53	0.002
	Time and group interaction term	0.28	0.07	<0.001
VAS pain^c^	Time	-0.05	0.01	<0.001
	Group (B50 vs. Α50)	-0.36	0.09	<0.001
	Time and group interaction term	0.02	0.01	0.048

**Figure 2:**
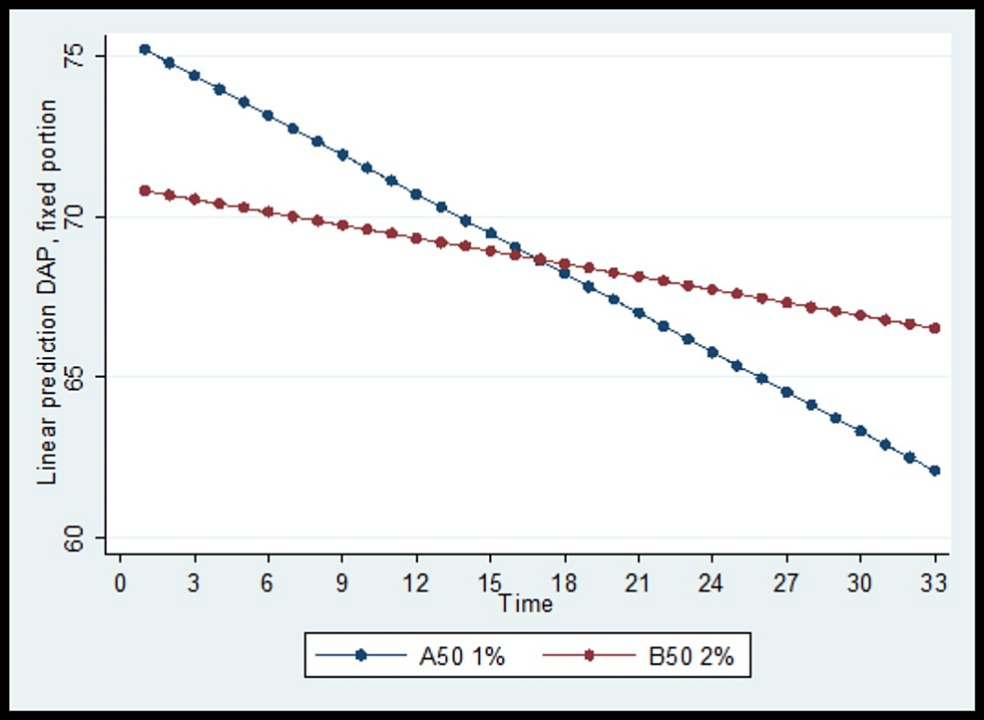
Diastolic arterial pressure (DAP) change over the follow-up period, by group

On the contrary, the baseline diastolic arterial pressure (DAP) was significantly lower in the B50 group. The DAP was reduced significantly throughout the follow-up period in both study groups, but the degree of reduction was greater in the A50 group (Figure 2).

**Figure 3:**
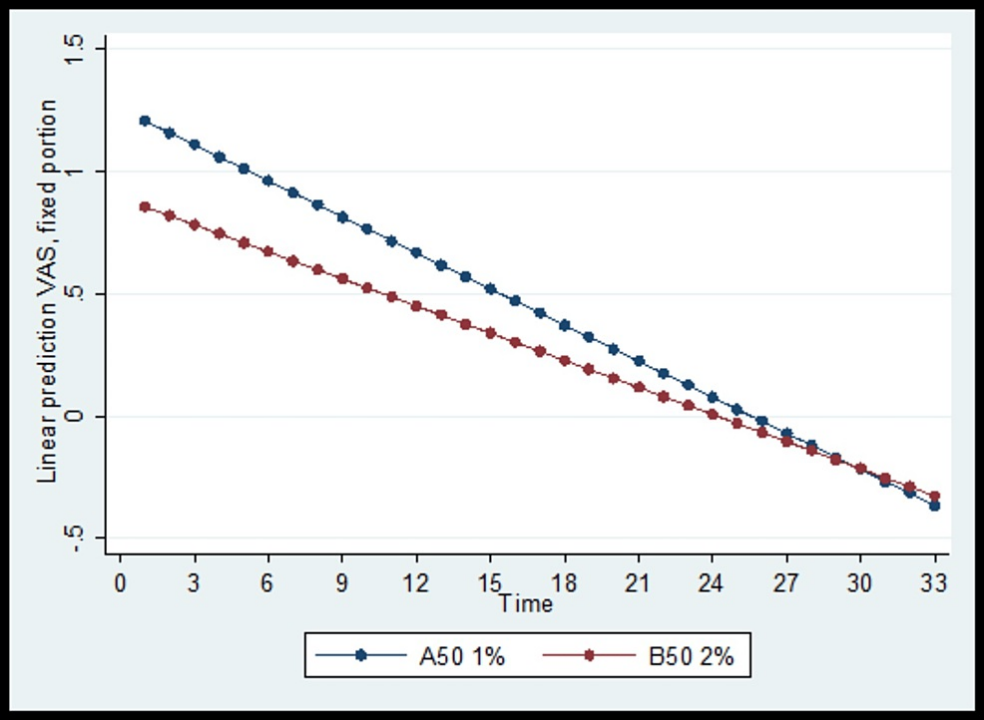
Visual analog scale (VAS) score changes over the follow-up period, by group

Similarly, the baseline VAS score was significantly lower in the B50 group and reduced significantly throughout the follow-up period in both study groups. Pain relief was significantly greater in the A50 group, as shown in Figure 3.

None of the patients experienced any adverse side effects, such as pruritus, shivering, headache, and limb numbness.

## Discussion

The primary endpoint of our investigation was motor blockade from the epidural administration of different concentrations of ropivacaine as assessed by the MBS. All the parturients had an MBS score of 6 (no motor blockade). This result comes in accordance with the findings of other authors that intermittent epidural dosing of local anesthetics produces less motor blockade than the continuous infusion [7,10,11], which is desirable in the setting of labor as is of great importance that the parturient is able to push sufficiently.

A secondary endpoint of our study was ropivacaine consumption. The number of doses requested by the women beyond the baseline boluses was significantly higher in the 0.1% concentration group, but overall consumption was almost equal. We presume that this can be attributed to the denser sensory block of the 0.2% ropivacaine solution. In addition, lower VAS scores were recorded by the parturients of the 0.2% ropivacaine group, which can be explained by the better quality that sensory blocks with higher ropivacaine concentration offer [12].

As far as instrumental delivery and conversion to cesarean section are concerned, there is no consensus among the experts. Sultan et al. [13] in a meta-analysis of 11 studies found that higher concentrations of local anesthetics increased the likelihood of assisted vaginal delivery. By contrast, Zhang et al. [14] in a 2021 review of nine studies concluded that there is no increased risk for assisted vaginal delivery and cesarean section with higher ropivacaine concentrations. Our study results support the latter findings, as the incidence of instrumental delivery and cesarean section did not differ between the two groups using different concentrations of ropivacaine.

It is well established that epidural can lead to sympathetic blockade and thus a reduction in blood pressure, especially in the pregnant population. The same dose of the local anesthetic has a wider spread in the epidural space due to its compression by the enlarged uterus [15]. This phenomenon is dose-dependent. An interesting finding in our study was the sole lower diastolic arterial pressure of the women receiving higher ropivacaine concentrations. It is common for clinicians to identify maternal hypotension as a drop in systolic arterial pressure [16], but diastolic arterial pressure may be of greater importance, especially for placental blood supply and labor outcomes [17,18].

Another outcome of the present study was the prolonged duration of the first stage of labor by the higher ropivacaine concentration solution. This is supported by a review by Callahan et al. [19] as epidural can cause prolongation of both the first and second stages. By contrast, Zhang et al. [14] found that a lower concentration of ropivacaine results in the prolongation of the first stage.

Neonatal status was also assessed in our study by recording APGAR scores at one, five, and 10 minutes. The group allocated to 0.2% ropivacaine concentration had significantly lower APGAR scores at the first minute, which cannot be attributed to the incidence of instrumental delivery and cesarean section. Many authors found no difference in APGAR scores with different ropivacaine concentrations [20-21], while others found more favorable scores with higher concentrations [11]. The feedback from the obstetricians regarding every delivery might explain such a result. The obstetricians observed that some women could not push as sufficiently as others. After the data analysis, this group of parturients was the one receiving 0.2% ropivacaine. We hypothesize that difficulty in maternal pushing resulted in fetal distress, which was the culprit of the lower APGAR scores. Maybe other factors such as the morbidities of the newborns could be responsible for such results, but these are beyond the scope of this study.

Furthermore, it is a common practice in our country for the anaesthesiologist to be present or make very frequent visits to the parturient who received an epidural in the labor room until delivery of the newborn, which has reduced personnel available for on-call emergencies. PIEBs seem to be the solution to this issue, as they appear to be a safe and satisfactory method for analgesia without any major disadvantages.

Our study has several limitations. Ropivacaine was provided by epidural pumps with specific dosage volume, time interval, and preset speed of delivery. Using different settings or pumps may result in different local anesthetic spread in the epidural space and thus different outcomes. Another limitation of our study is that we recorded only the motor blockade caused by different concentrations of ropivacaine but not the patient satisfaction, which is a parameter that plays a significant role in the labor experience of the parturient and should be recorded in future studies. Lastly, obstetrician feedback on maternal pushing was not quantified, which renders it challenging to compare the difficulty of maternal pushing between clinicians.

## Conclusions

In summary, we concluded that both concentrations of ropivacaine used for labor analgesia with the PIEB mode provide excellent analgesia and satisfaction for the expectant mother without any motor blockade. However, higher concentrations, such as 0.2% ropivacaine, may lead to some unfavorable results for both the parturient and the newborn, such as lower diastolic blood pressure, prolonged duration of labor, lower APGAR score of the newborn, and less satisfaction for the obstetrician during the extrusion stage that translates to inadequate pushing. In our hospital, 0.1% instead of 0.2% ropivacaine will be implemented in our protocols in the future. More studies need to be conducted for the ideal concentration of local anesthetics using the PIEB method as it seems to be a reliable and safe method of administering analgesia to the parturient.
